# KRAS Activation and over-expression of SIRT1/BCL6 Contributes to the Pathogenesis of Endometriosis and Progesterone Resistance

**DOI:** 10.1038/s41598-017-04577-w

**Published:** 2017-07-28

**Authors:** Jung-Yoon Yoo, Tae Hoon Kim, Asgerally T. Fazleabas, Wilder A. Palomino, Soo Hyun Ahn, Chandrakant Tayade, David P. Schammel, Steven L. Young, Jae-Wook Jeong, Bruce A. Lessey

**Affiliations:** 1 Obstetrics, Gynecology & Reproductive Biology, Michigan State University, Grand Rapids, MI 49503 USA; 2 Department of Biochemistry and Molecular Biology, Yonsei University College of Medicine, Seoul, 03722 South Korea; 3 Department of Women’s Health, Spectrum Health System, Grand Rapids, MI 49341 USA; 4 Institute for Maternal and Child Research, Faculty of Medicine, University of Chile, Santiago, Chile; 5 Department of Biomedical and Molecular Sciences, Queens University, Kingston, ON K7L 3N6 Canada; 6 Pathology Associates, Greenville Hospital System, Greenville, SC 29605 USA; 7 Obstetrics and Gynecology, University of North Carolina, Chapel Hill, NC 27514 USA; 8 Obstetrics and Gynecology, Greenville Health System, Greenville, SC 29605 USA

## Abstract

Endometriosis is an inflammatory condition that is associated with progesterone resistance and cell proliferation, resulting in pain, infertility and pregnancy loss. We previously demonstrated phosphorylation of STAT3 in eutopic endometrium of infertile women with this disorder leading to over-expression of the oncogene BCL6 and stabilization of hypoxia-induced factor 1 alpha (HIF-1α). Here we report coordinated activation of KRAS and over-expression of Sirtuin 1 (SIRT1), a histone deacetylase and gene silencer, in the eutopic endometrium from women with endometriosis throughout the menstrual cycle. The mice with conditional activation of KRAS in the PGR positive cells reveal an increase of SIRT1 expression in the endometrium compared to control mice. The expression of progesterone receptor target genes including the Indian Hedgehog pathway genes are significantly down-regulated in the mutant mice. SIRT1 co-localizes with BCL6 in the nuclei of affected individuals and both proteins bind to and suppress the promoter of *GLI1*, a critical mediator of progesterone action in the Indian Hedgehog pathway, by ChIP analysis. In eutopic endometrium, GLI1 expression is reduced in women with endometriosis. Together, these data suggest that KRAS, SIRT1 and BCL6 are coordinately over-expressed in eutopic endometrium of women with endometriosis and likely participate in the pathogenesis of endometriosis.

## Introduction

Endometriosis is a gynecologic disorder defined by the presence of endometrial cells outside of the uterine cavity. Endometriosis adds significantly to health care costs, upwards of $22 billion dollars per year in the US^1^. Symptoms of endometriosis vary widely and include dysmenorrhea, dyspareunia, noncyclic chronic pelvic pain, and infertility, with a considerable negative impact on quality of life^1, 2^. Endometriosis is associated with both infertility and pelvic pain and affects about 5% of reproductive-age women and up to 50% of these are infertile^3, 4^. Surgical removal of ectopic lesions and/or hormonal suppression focused on reducing estrogen, such as progestins, androgens, gonadotropin-releasing hormone (GnRH) agonists, and aromatase inhibitors are the current gold standards of therapy. However, both approaches are associated with various side effects and a high incidence of relapse^5^. Therefore, identification of mechanisms involved in the pathogenesis of endometriosis and strategic therapies for treatment remain critical.

The eutopic endometrium of women with endometriosis exhibits inflammation, aberrant estrogen activity, cellular proliferation and a resistance to progesterone (P4)^5^. The biological mechanisms linking endometriotic lesions to these endometrial alterations remains uncertain and controversial, while P4 resistance and estrogen dominance likely contributes to the pathophysiology and survival of ectopic lesion and contributes to infertility^6, 7^. KRAS has been proposed as a strong candidate gene in the pathophysiology of endometriosis. Activation of KRAS in mice was associated with endometriosis-like lesions on the peritoneum and ovaries^8^ and lesions derived from mice with activated KRAS mutation survived longer in wild type mice^9^. While there is no direct link between KRAS mutations and the risk for endometriosis in humans^10^, inflammation associated events including changes in miRNA expression in endometriosis^11^, may play a role in its activation^12^. We previously showed that miRNA34b was dramatically decreased in eutopic endometrium of women with endometriosis^13^. This miRNA has been shown to have benefit in KRAS induced mouse models of other carcinomas^14^. Both let-7b and miRNA 34 have been shown to target KRAS^15^, and both miR34 and p53 can act synergistically to suppress tumor growth^16^.

BCL6 (B Cell Lymphoma 6) is a transcriptional gene repressor and is necessary for B cell development and oncogenesis^17^. BCL6 has six Krüppel-type zinc finger domains and a BTB/POZ (bric-á-brac, tramtrack, broad complex/pox virus zinc finger) domain, which can bind to transcriptional factors, including Interferon Regulatory Factor (IRF) 4 and BCL6-associated zinc finger (BAZF)^18^. BCL6 is one of the human proto-oncogenes and is associated with an increase in cell proliferation through the repression of genes such as p53 and p300^19^. BCL6 DNA binding site (TTCCT(A/C)GAA) is similar with Signal Transduction and Activators of Transcription (STAT) factors and BCL6 can repress transcription via STAT factor binding sites and thus inhibit cytokine-induced transcription^20^. Furthermore, BCL6 is up-regulated by STAT 3^21^. STAT3 signaling is aberrantly activated in eutopic endometrium from women with endometriosis compared to those without this disease^22^. Recently, we reported that BCL6 is highly over-expressed in endometrium from women with endometriosis during the secretory phase of the menstrual cycle compared to women without endometriosis^23^.

SIRT1 is a member of the sirtuin family of proteins and homologs to the yeast Sir2 protein. Sirtuin family proteins are Class III HDACs^24^. SIRT1 can deacetylate both histones and non-histone proteins such as p53^25^. Its deacetylation activity enables it to regulate gene transcription and implicates the influence of a variety of cellular processes such as aging, apoptosis, inflammation, stress resistance, and metabolism^26^. SIRT1 has a dual role as oncogenic function as well as tumor suppressor^27^. According to previous reports, SIRT1 plays a role as a tumor promoter in endometrial cancer by targeting sterol regulatory element binding protein 1 (SREBP1) and lipogenesis^28^. Additionally, SIRT1 has an important role in the regulation of inflammatory cytokines expression in endometriotic stromal cells^29^ and SIRT1 has been associated with poor prognosis ovarian cancers^30^. However, the role of SIRT1 in endometriosis and uterine biology has not been examined.

In this study, we investigated the levels of KRAS and SIRT1 proteins in eutopic endometrium from women with endometriosis. The levels of SIRT1 and KRAS were compared in endometrium of women with and without endometriosis. Using a mouse model, we investigated the potential link between KRAS activation and SIRT1 expression. We report here for the first time in endometrium, direct protein-protein interactions between SIRT1 and BCL6 in human endometrial tissue, co-localizing in the nuclei of endometriosis cases. Further, we show that GLI1, a promoter target for both BCL6 and SIRT1, is reduced in eutopic endometrium of women with this disease. Based on these results we suggest that aberrant overexpression of SIRT1 is driven by KRAS activation, and co-localizes with BCL6 contributing to the phenomenon of P4 resistance through gene inactivation of the *GLI1* promoter.

## Results

### Endometriosis and Inflammation

**Figure 1:**
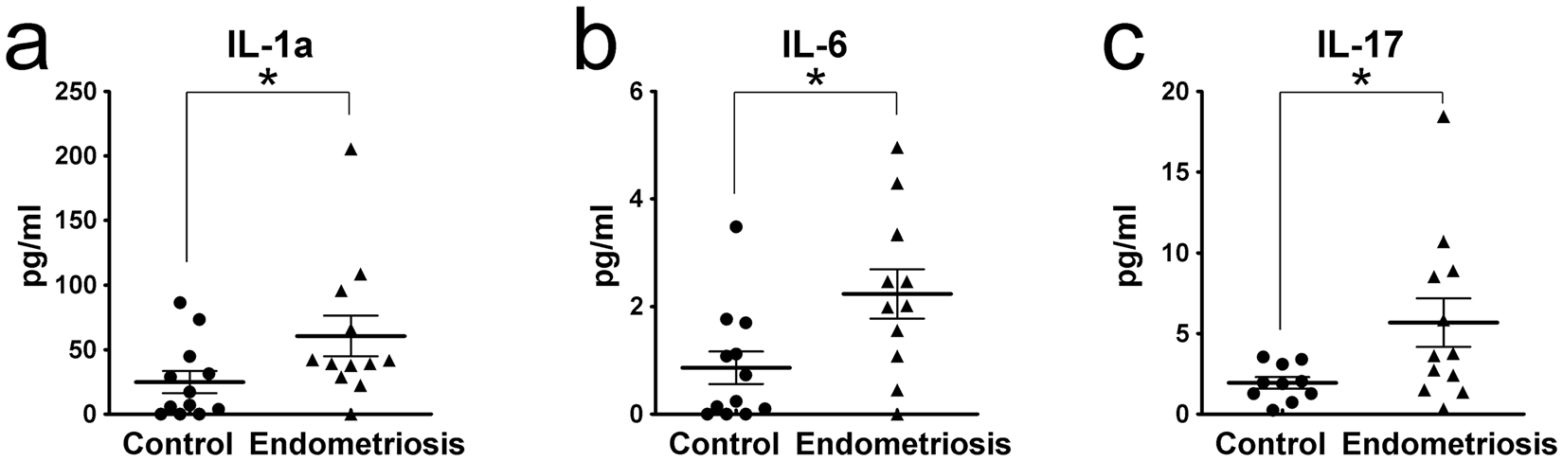
Endometriosis is associated with elevated serum inflammatory cytokines including (**a**) IL-1a, (**b**) IL-6 and (**c**) IL-17, compared to normal controls.

Endometriosis is the presence of glands and stroma outside the uterus. It is often found on the ovary and peritoneum. Endometriosis is a chronic inflammation disease. To better understand the systemic inflammation status of endometriosis patients, we measured inflammatory cytokines in plasma of women with and without endometriosis using a multi-plex array from Eve Technologies. Our results revealed significant elevations in IL-1a, IL-6 and IL-17, among others (Fig. 1).

### Overexpression of KRAS and SIRT1 in eutopic endometrial tissue from women with endometriosis

**Figure 2:**
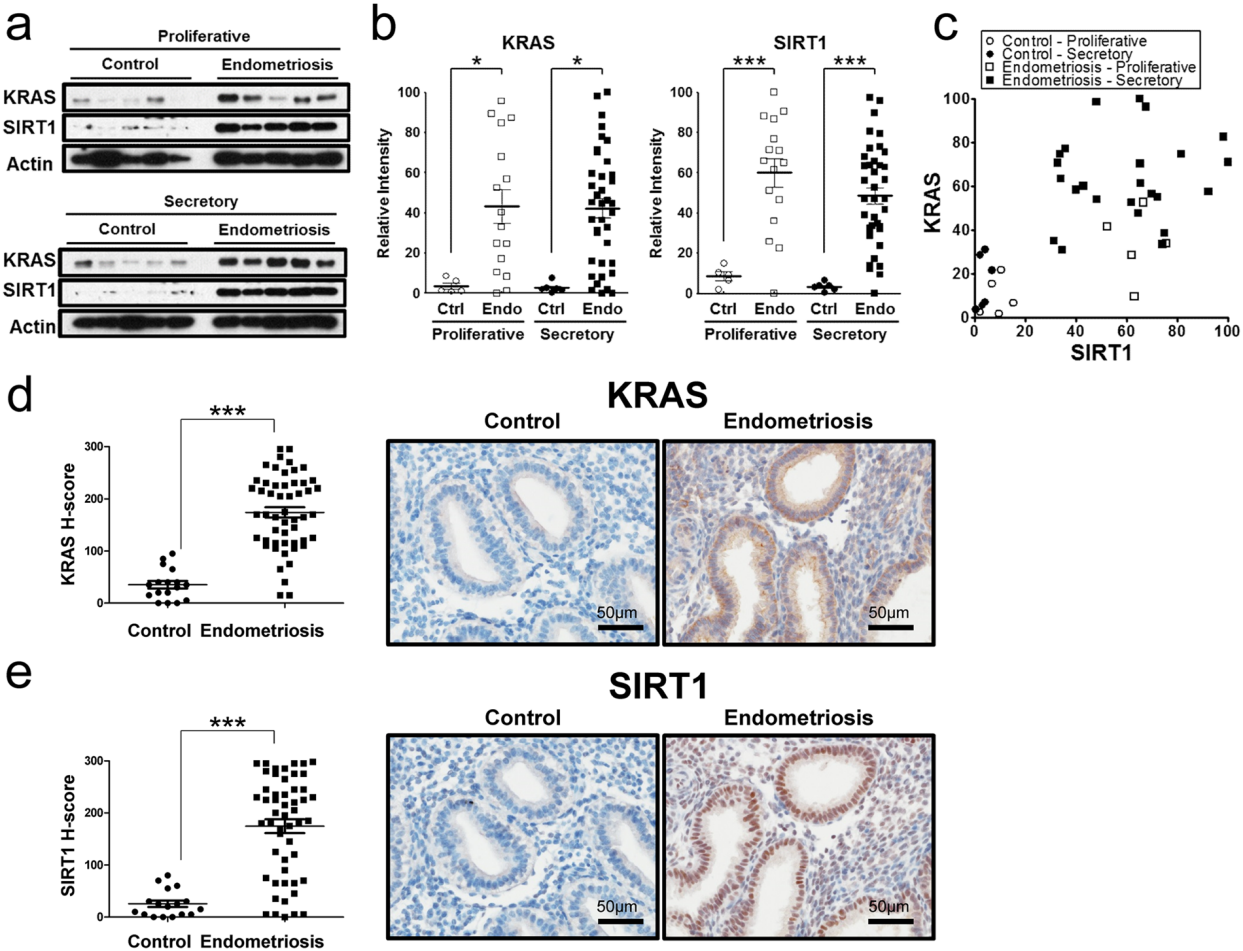
Correlation of between KRAS and SIRT1 in human endometrium with endometriosis. (**a**) Western blot analysis of SIRT1 and BCL6 proteins in proliferative and secretory phases of human endometrium with endometriosis. β-actin was used as sample-loading control. Representative blots have been cropped to reduce unnecessary area. Full-length blots are presented in Supplementary Fig. S2. (**b**) Densitometric analysis of KRAS and SIRT1 protein levels by Western blot analysis in eutopic endometrium from proliferative and secretory phase in women with and without endometriosis. (**c**) Correlation between SIRT1 and KRAS in women with endometriosis throughout the menstrual cycle phases based on Western blot analysis (correlation coefficient = 0.4641, p = 0.0009). (**d** and **e**) H-score of KRAS (**d**) and SIRT1 (**e**) expression in endometrium from women with and without endometriosis and representative photomicrograph of immunohistochemical staining of KRAS in endometrium from women without and with endometriosis. The results represent the mean ± SEM. ***p < 0.001.

IL-6 activates JAK kinases and Ras-mediated signaling. Activation of KRAS, the key regulator of Ras/ERK pathway, in the endometrium of mice causes ectopic lesion establishment^9^. KRAS appears to be dysregulated in endometriosis. To document this, we first examined the expression of KRAS in endometrium using Western blot, using women with and without the disease. While the expression levels of KRAS did not differ between proliferative (n = 21) and secretory phase (n = 44), the levels of KRAS were significantly higher in the endometrium that originated in subjects with endometriosis (n = 54) as compared to controls (n = 11) (Fig. 2a and b). KRAS activation is associated with overexpression of SIRT1^31^. KRAS activation regulates epithelial-mesenchymal transition and cell migration through SIRT1^32^. To better understand the finding of increased KRAS in the endometrium of women with endometriosis, we investigated the association between KRAS activation and SIRT1 expression. The levels of SIRT1 protein were significantly increased in the samples from women with endometriosis (n = 54) compared with controls (n = 11) (Fig. 2a and b). However, SIRT1 expression was low and unchanged during the menstrual cycle in the control group. Figure 2c showed a significant positive correlation between KRAS and SIRT1 proteins in the endometrium of the control and endometriosis group (Spearman correlation coefficient r = 0.6155, p < 0.0001).

To determine the cell-specific expression of KRAS and SIRT1, we performed immunohistochemical analysis in endometrium from women with and without endometriosis (Fig. 2d and e). In control women KRAS and SIRT1 proteins were weakly detected in the stromal and epithelial cells of endometrium from the proliferative phase and early, mid, and late secretory phases in women without endometriosis (n ≥ 4 per phase) (Supplementary Fig. S1). Interestingly, the levels of KRAS protein were significantly increased in the stromal and epithelial cells of endometrium from proliferative and secretory phase endometriosis patients (n = 52) as compared to control patients (n = 17) (Fig. 2d). The levels of SIRT1 were also significantly higher in both the stromal and epithelial cells of endometriosis patients compared to women without endometriosis (Fig. 2e). These results argue that aberrant activation of KRAS and SIRT1 is integral to pathogenesis of endometriosis. In addition, like BCL6, they appear to be specific endometrial biomarkers for the diagnosis of endometriosis.

### Correlation between SIRT1 and BCL6 in endometriosis

**Figure 3:**
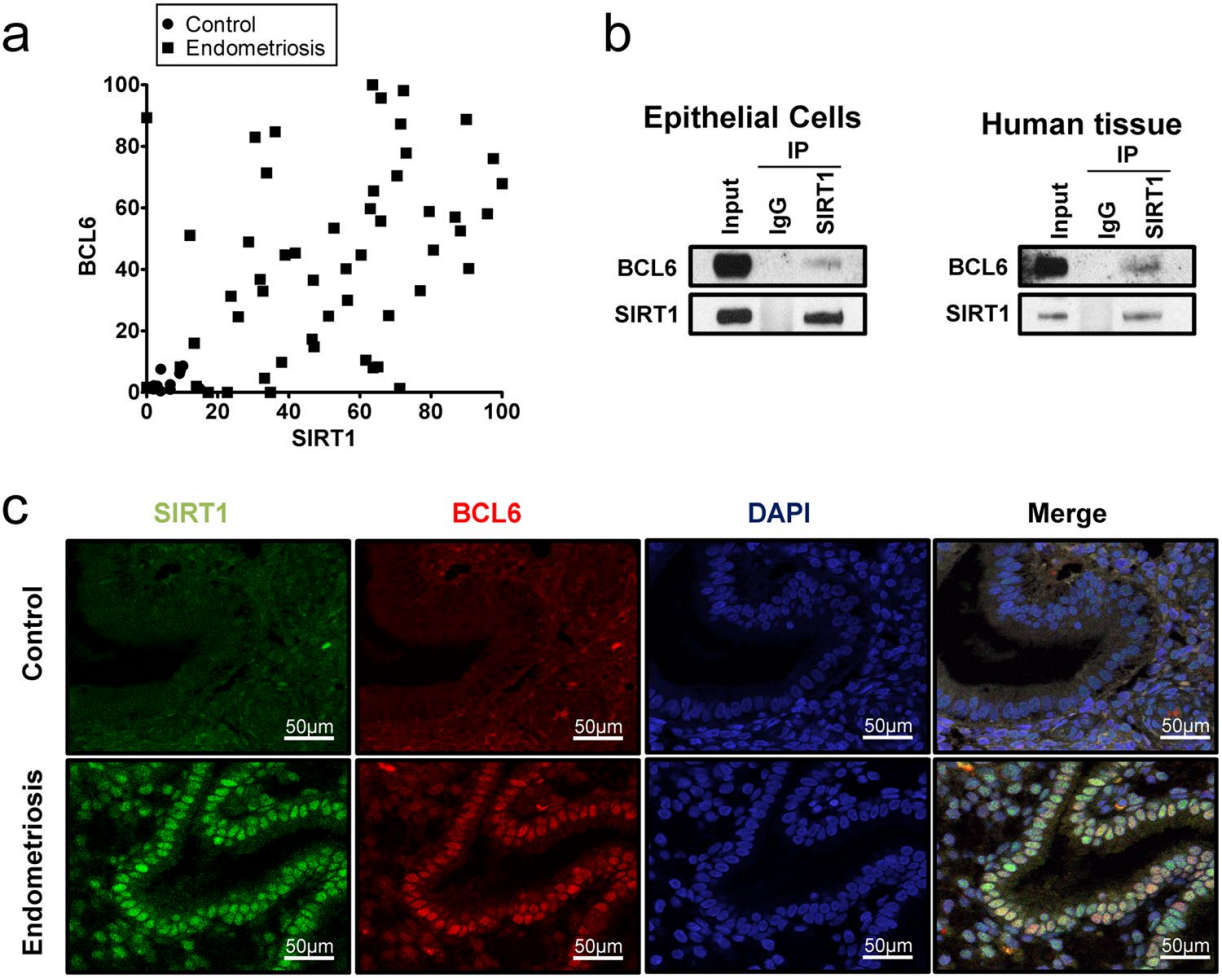
Correlation of between SIRT1 and BCL6. (**a**) Correlation analysis between SIRT1 and BCL6 in human endometrium with endometriosis. (**b**) Immunoprecipitation (IP) analysis between SIRT1 and BCL6 in Ishikawa cells and Human endometrium with endometriosis. Representative blots have been cropped to reduce unnecessary area. Full-length blots are presented in Supplementary Fig. S4. (**c**) Co-localization of SIRT1 and BCL6 in the human endometrium without and with endometriosis by immunofluorescence analysis.

BCL6 is a transcriptional repressor involved in B cell development and oncogenesis and known to be involved in the recruitment of SIRT1 deacetylase^33^. We previously reported over-expression of BCL6 in eutopic endometrium of infertile women with endometriosis^23^. Since both proteins appear to be elevated, we analyzed the relationship between SIRT1 and BCL6 proteins in eutopic endometrium of endometriosis patients. The levels of SIRT1 and BCL6 were examined and compared in eutopic endometrium using Western blot analysis. Our results showed a strong positive correlation between SIRT1 and BCL6 levels in women with endometriosis throughout the menstrual cycle phases (n = 44). Based on the Western blot band intensity, we show a scattergram with a correlation coefficient = 0.5659, p < 0.0001, between BCL6 and SIRT1 expression (Fig. 3a).

To determine whether SIRT1 physically interacts with BCL6, we performed immunoprecipitation with SIRT1 antibody in total protein lysates from Ishikawa human endometrial adenocarcinoma cell line and endometrium from endometriosis patients. The immunoprecipitation result showed that endogenous SIRT1 physically interacts with BCL6 in human endometrium (Fig. 3b). However, no BCL6 was detected within the immune-precipitate of the IgG negative control. To determine whether SIRT1 proteins co-localize with BCL6 proteins, we performed double immunofluorescence for SIRT1 and BCL6. The immunofluorescence results show that SIRT1 and BCL6 proteins were co-localized in endometrial epithelial cells of endometriosis patients (Fig. 3c). These finding leads to the conclusion that these proteins may be acting in concert and contribute to the development or maintenance of endometriosis.

### Aberrant activation of SIRT1 and BCL6 expression in a baboon model of endometriosis progression

**Figure 4:**
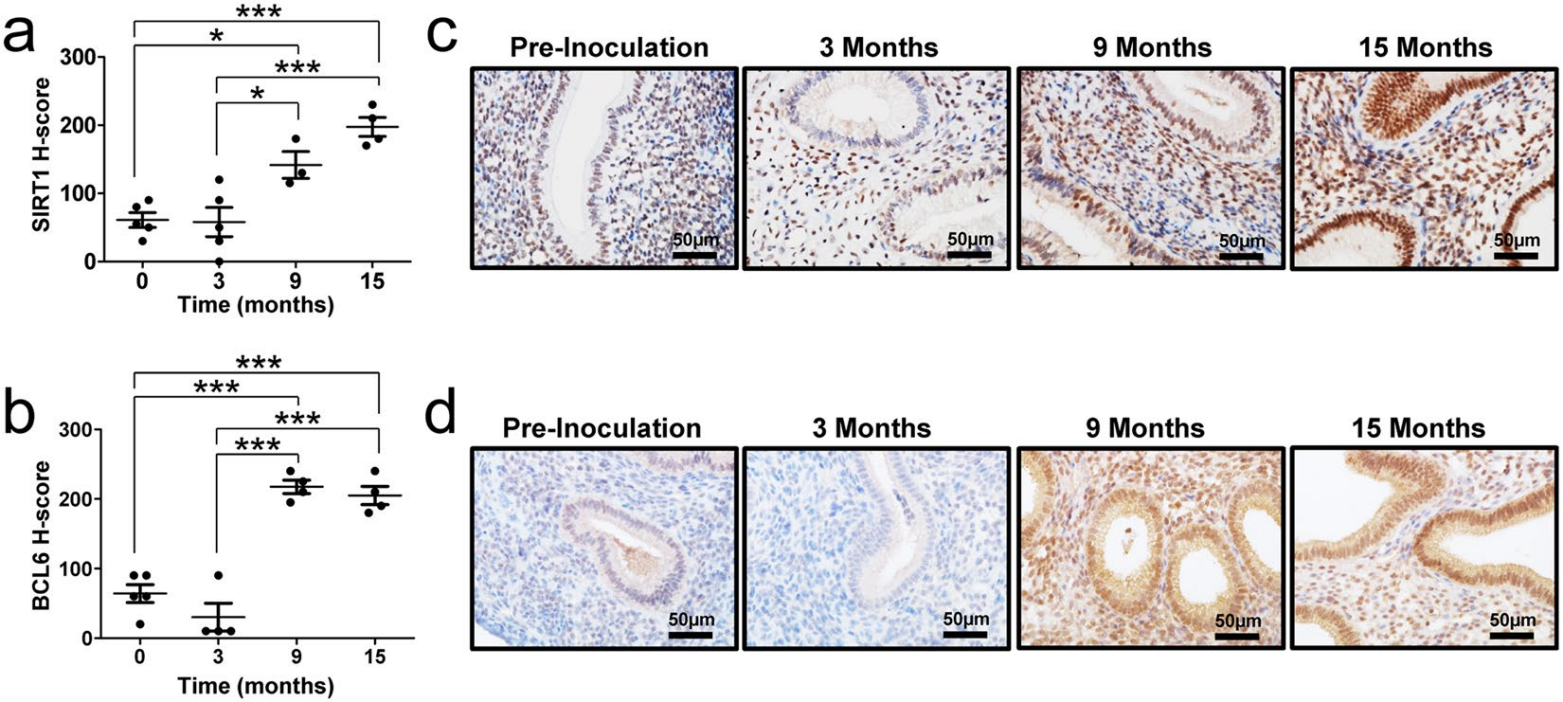
Levels of SIRT1 and BCL6 proteins during progression of endometriosis in a baboon model. (**a** and **b**) H-score of SIRT1 (**a**) and BCL6 (**b**) expression in endometriosis baboon model induced by intraperitoneal inoculation of menstrual endometrium during progression of endometriosis. The results represent the mean ± SEM. *p < 0.05 and ***p < 0.001. (**c** and **d**) Representative photomicrograph of immunohistochemical staining of SIRT1 (**c**) and BCL6 (**d**) in the baboon endometrium of pre-inoculation and 3, 9 and 15 months post-inoculation during endometriosis progression.

We have used primate models to study temporal sequence of events involved in endometriosis establishment and progression^34^. To determine that SIRT1 and BCL6 proteins are overexpressed as part of endometriosis development, we performed immunohistochemical analysis of SIRT1 and BCL6 in eutopic baboon endometrium sequentially after the experimental induction of the disease (n = 4 per time point). As in human endometrium, the expression of SIRT1 and BCL6 proteins were not evident in the endometrium of pre-inoculation (control) baboons. The levels of SIRT1 and BCL6 proteins were significantly increased at 9 and 15 months post-inoculation during endometriosis progression (Fig. 4). These data suggest that the ontogeny of BCL6 and SIRT1 expression occur synchronously, and that they require time after initiation of endometriosis to develop. The timing of the appearance of SIRT1 and BCL6 corresponds to the increase in inflammation seen in this model.

### SIRT1 overexpression and dysregulation of PGR target genes in mice with uterine specific KRAS activation

**Figure 5:**
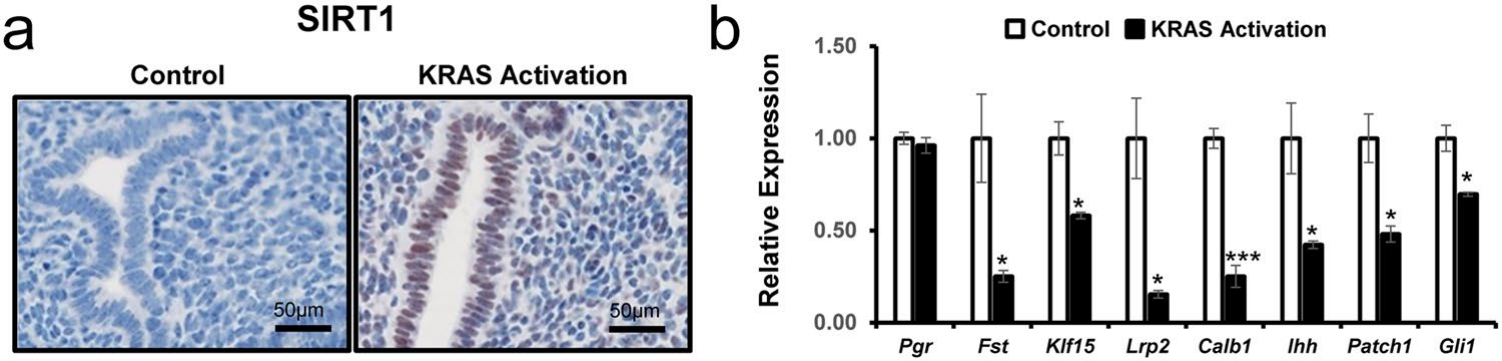
Levels of SIRT1 in the KRAS activation mouse model. (**a**) Representative photomicrograph of immunohistochemical staining of SIRT1 in the control and KRAS activation mouse. (**b**) The mRNA expression level of P4 target genes in the uterus from control and KRAS activation mice (n = 9). The results represent the mean ± SEM. *p < 0.05 and ***p < 0.001.

In order to effectively investigate the effects of KRAS activation in endometrium, mice with loxP-Stop-loxP-Kras^*G12D*/+^ (*LSL-K-ras* ^*G12D*/+^)^35^ were bred to the PGR^Cre^ mouse (*Pgr* ^*cre*/+^ *LSL-K-ras* ^*G12D*/+^ mouse)^36^. Introduction of the oncogenic *K-ras* mutation in all PGR-positive cells did not show any pathological phenotype in the uterus^37^. We investigated whether KRAS activation altered the expression of SIRT1 in the mouse uterus using immunohistochemistry (n = 3 per group). Interestingly, SIRT1 expression was highly increased in endometrium of the mutant mice compared to control mice (Fig. 5a). We performed real-time RT-PCR to assess the expression of PGR and its target genes in the mutant mice. *Pgr* expression was not changed in the mutant mice. The mRNA expression level of P4 target genes, *Fst*, *Klf15, Lrp2*, and *Calb1*, were highly downregulated in the mutant mice compared to the control mice. Interestingly, the expression of *Ihh*, *Patch1*, and *Gli1* which are known as P4-target and Indian Hedgehog pathway genes were significantly downregulated in the mutant mice (Fig. 5b). These results suggest that KRAS suppresses transcriptional activity of PGR by regulating SIRT1 expression.

### Transcriptional Repression of GLI1 by SIRT1 and BCL6 proteins

**Figure 6:**
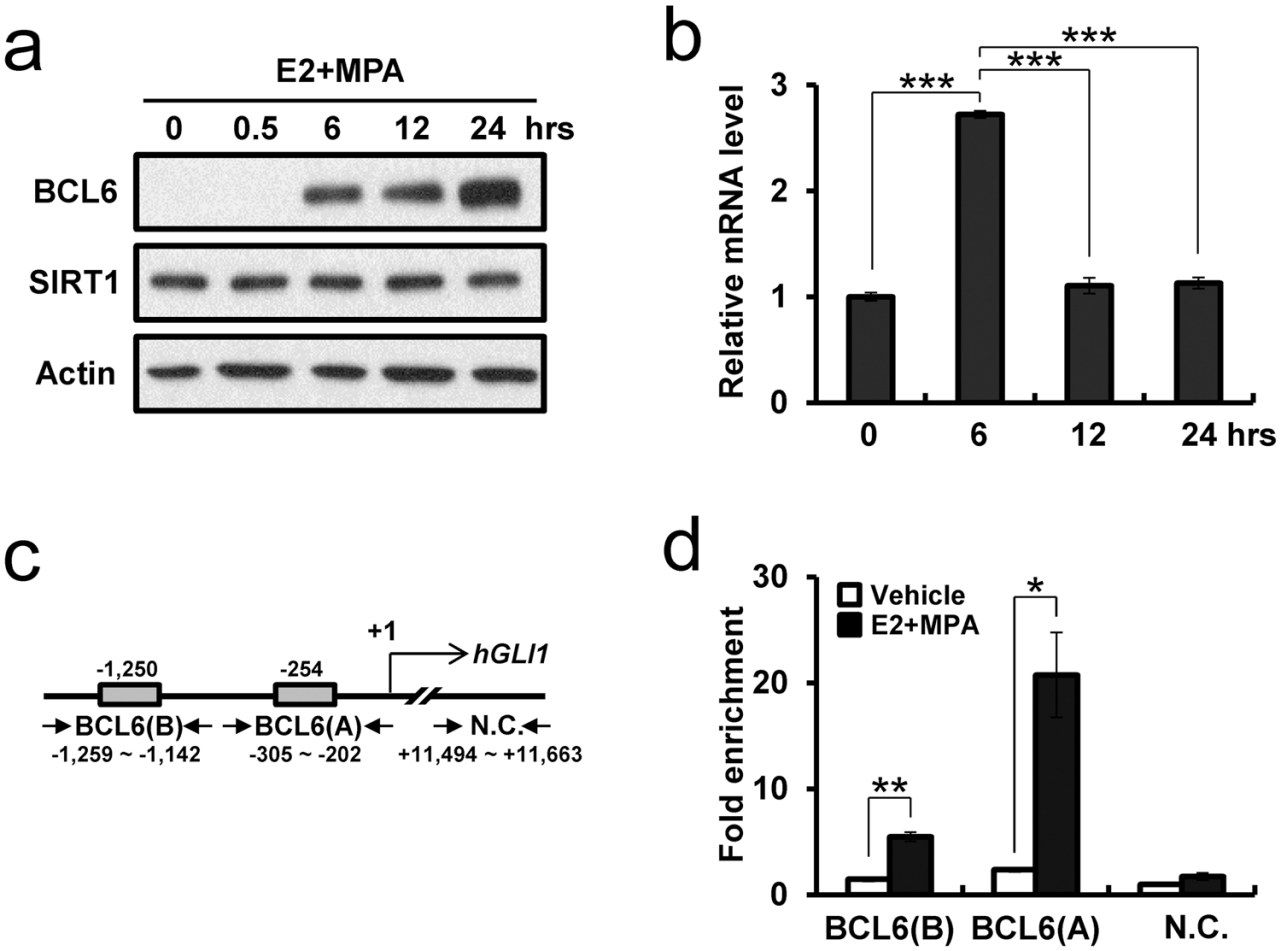
Regulation of GLI1 gene expression by SIRT1 and BCL6 proteins. (**a**) Western blot analysis of BCL6 and SIRT1 in Ishikawa cells treated with E2 + MPA for 0, 30 min, 6, 12, and 24 hours. β-actin was used as sample-loading control. Representative blots have been cropped to reduce unnecessary area. Full-length blots are presented in Supplementary Fig. S5. (**b**) Quantitative real time PCR analysis of *GLI1* gene expression in Ishikawa cells treated with E2 + MPA for 0, 6, 12, and 24 hours. (**c**) Map of BCL6 binding site on the *GLI1* promoter (Gray boxes). Negative control (N.C.) region on the *GLI1* gene was used as negative control of ChIP assay. Primers used in ChIP assay are presented by arrows. (**d**) ChIP assay using anti-SIRT1 antibody on *GLI1* promoter in Ishikawa cells treated with or without E2 + MPA for 24 hours. The results represent the mean ± SEM. *p < 0.05, **p < 0.01, and ***p < 0.001.

E2 stimulates proliferation of uterine epithelial cells while P4 is inhibitory to E2-mediated proliferation of the epithelium^38, 39^. The major pathologic phenomenon of uterine disease is the loss of ovarian steroid hormone control over uterine epithelial cell proliferation and apoptosis^40–43^. Resistance to P4 treatment, via loss of progesterone receptors (PGR) or its signaling pathways, is a major hurdle in the treatment of a variety of diseases in the endometrium of women such as endometriosis and endometrial cancer^36, 44–46^. To gain insight into the underlying molecular mechanisms of SIRT1/BCL6 action in an epithelial cell model of endometrium, Ishikawa cells were treated with E2 + MPA and subsequently used Western blot analysis to examine the expression levels of BCL6 and SIRT1. The level of BCL6 was increased gradually after 6 hours by E2 + MPA (Fig. 6a). SIRT1 levels were consistently strong in Ishikawa cells. Interestingly, the expression of *GLI1* was significantly decreased after 12 hours treated with E2 + MPA (Fig. 6b). These results suggest that E2 + MPA, a known inducer of BCL6, results in repression of GLI1 expression.

To determine that BCL6 and SIRT1 bind to the putative *GLI1* promoter, we performed ChIP analysis on chromatin from Ishikawa cells treated with E2 + MPA. Our ChIP results exhibited that both BCL6 and SIRT1 proteins were significantly accumulated on two sites (BCL6 (A) and (B)) of *GLI1* promoter in Ishikawa cells treated with E2 + MPA compared to vehicle control. Interestingly, the accumulated SIRT1 protein closely parallels what BCL6 protein accumulated on *GLI1* promoter (Fig. 6c and d). These results suggest that BCL6 regulates transcriptional repression of *GLI1* expression through direct interaction with SIRT1 in endometrial epithelial cells. Further, elevated levels of SIRT1 and BCL6 in secretory phase endometrium of women with endometriosis likely accounts for the decrease noted in GLI1 protein expression, as a sign of P4 resistance.

### Attenuation of GLI1 expression in endometrium from women with endometriosis

**Figure 7:**
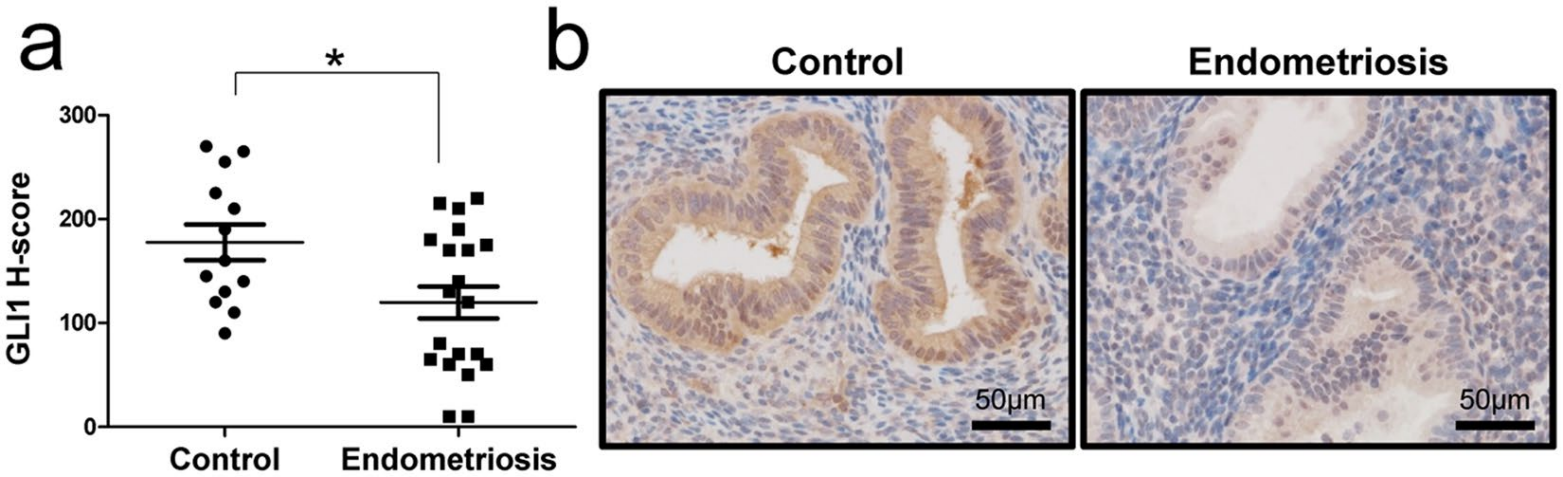
Levels of GLI1 in endometrium from women with and without endometriosis. (**a**) H-score of GLI1 expression in endometrium from women with and without endometriosis. The results represent the mean ± SEM. *p < 0.05. (**b**) Representative photomicrograph of immunohistochemical staining of GLI1 in endometrium from women without and with endometriosis.

SIRT1/BCL6 proteins act as a transcriptional repressor of *GLI* effectors in the Hedgehog pathway for neurogenesis and tumor suppression of medulloblastoma^33^. Therefore, we examined GLI1 expression in eutopic endometrium from women with (n = 20) and without (n = 13) endometriosis by immunohistochemistry. Our immunohistochemistry analysis found that GLI1 protein levels are significantly reduced, specifically in the endometrial epithelial cells comparing women with and without endometriosis (Fig. 7).

## Discussion

KRAS, a well characterized oncogene, has been implicated in the pathogenesis of endometriosis^12^. While mutational changes to KRAS appears to be a pivotal change in endometriosis-related ovarian cancers^47^, we demonstrate for the first time, that KRAS activation is a common finding and a key biomarker in the endometrium of most women with endometriosis. We show that its activation is highly correlated to the over-expression of SIRT1 (member of the sirtuin family) and contributes to the upregulation of this histone deacetylase. Further, we postulate that inflammatory changes associated with endometriosis provide the milieu for activation of KRAS mediated BCL6/SIRT1 complexes that participate in the early stages of P4 resistance, which contributes to infertility and a key to the pathophysiology of endometriosis growth and pathogenesis^48^.

In the present study, we report that BCL6 and SIRT1 are over-expressed and co-localize in the nuclei of endometrial cells from women with endometriosis. SIRT1 is a nicotinamide adenosine dinucleotide (NAD)-dependent deacetylase that is responsible for a wide variety of vital functions in the cell by removing acetyl groups from histone and non-histone proteins controlling gene expression^49^. While the regulatory controls for endometrial SIRT1 remain unknown, BCL6 is up-regulated by inflammatory stimuli including IL-6 and STAT3 activation^50^, that we recently examined^22^. The number of known SIRT1 targets are many and include genes involved in endometrial function and P4 action, including GLI1, FOX01, PPARγ, CTIP2 (chicken ovalbumin upstream promoter transcription factor interacting protein 2 (COUP-TFII), and p300^51^.

SIRT1 has been shown to pair with other transcription factors including BCL6. BCL6 is a transcriptional repressor involved in B cell development and oncogenesis^52^. We showed that BCL6 is over-expressed in eutopic endometrium of women with endometriosis^53^. We report for the first time that BCL6 and SIRT1 interacting through the IHH pathway both bind to and inactivate the GLI1 promoter. Tiberi *et al*. had reported similar findings in the Sonic Hedgehog pathway, showing that BCL6/BCOR/SIRT1 complex suppresses growth of human medulloblastoma cells through *GLI1* and *GLI2* repression^33^. Collectively, these data suggest thatBCL6/SIRT1 could influence chromatin acetylation patterns at the *GLI1*regulatory regions and thereby contribute to epigenetic repression of GLI1^54^. Identification of these BCL6/SIRT1-recruiting factors and the mechanism of protein-protein interaction will be of importance in future investigations.

The concept of P4 resistance in endometriosis is now well-established^55, 56^, though the underlying mechanism has remained elusive. Several mechanisms of cellular resistance to P4 have been suggested including alterations in progesterone receptor chaperone proteins FKBP52^57–63^, progesterone receptor coactivator Kruppel-like factor 9 (KLF9)^64^, MIG-6 alterations^65^, progesterone coactivator Hic-5^66^, and direct alterations of PGR subunits^67^. We believe SIRT1/BCL6 represent a more proximal defect in endometrium of women with endometriosis that interferes with early signaling of P4. As recently reviewed^68^, P4 initiates a complex series of paracrine signaling steps involving the Indian Hedgehog (IHH) expression by endometrial epithelium. GLI1 has been shown to play an integral role in this pathway^69–72^.

In this study, we demonstrated for the first time that SIRT1 is over-expressed in women with endometriosis compared to controls by western blot and immunohistochemistry, correlating directly with elevated BCL6 expression. Co-localization using immunofluorescence and co-immunoprecipitation confirmed direct interaction of SIRT1 with BCL6 in the nucleus of affected individuals. Perhaps most striking was the concurrent up-regulation of both proteins in baboon model of endometriosis, both BCL6 and SIRT1 appearing within 9 months of induction of the disease. Animal models are useful for studying the temporal sequence of events involved in disease establishment and progression. Autologous inoculation of autologous menstrual blood establishes endometriotic lesions that are histological and morphological similar to human disease. Together, these data support an inflammatory-driven phenomenon. Interestingly, BCL6 appears to be regulated by different pathways.

We show that IL-6 as well as other inflammatory mediators are higher in women with endometriosis. In P4 resistance, the normally repressive effect of STAT5 on BCL6 appears to be reduced, while the activation of STAT3 seen in endometriosis^22^ drives BCL6 over-expression^50^. SIRT1, on the other hand, is regulated by other factors. Estrogen has been shown to increase SIRT1^73^, as well as inflammation-driven miRNAs^74^. miRNA34 has been shown to inhibit SIRT1^75^ and we previously reported that miR34 levels are markedly reduced in women with endometriosis^13^, likely regulated by inflammation^76^. Thus, both SIRT1 and BCL6 over-expression can be regulated through inflammatory cytokines known to be present in women with endometriosis.

Furthermore and importantly, we show that KRAS activation in the mouse uterus is associated with increased SIRT1 proteins and suppressed expression of P4 target genes including Indian hedgehog pathway genes. P4 resistance implies a decreased responsiveness of target tissue to bioavailable P4^77^, and such an impaired P4 response is seen in the endometrium of women with endometriosis^78^. P4 resistance is associated with early secretory phase deficiency, early pregnancy loss, or infertility due to endometriosis. Understanding the molecular mechanisms of P4 resistance is critical to developing better therapeutic approaches to infertility and endometriosis. Therefore, our results suggest KRAS activation causes P4 resistant through SIRT1 in endometrium.

In summary, this is the first time that non-mutated KRAS activation has been shown to be strongly correlated with endometrium-associated endometriosis and that this activation triggers specific changes in histone deacetylase, SIRT1 which we postulate is a key driver of P4 resistance. SIRT1 is highly expressed in the endometrium of patients with endometriosis and appears to be an excellent biomarker in endometrium of women with this disorder. Transcriptional repression of GLI1 relies on recruitment of SIRT1 and BCL6 onto the promoter. These studies identify a primary mechanism of inflammatory dysfunction that contributes to the pathogenesis of endometriosis and may have a role in infertility and pregnancy loss associated with this disease. A lack of defined pathways has hampered the development of targeted pharmacological approaches that might benefit women with this disease. These novel findings regarding coordinated expression of SIRT1 and KRAS and the colocalization of BCL6 and SIRT1may improve treatment options for this enigmatic disease.

## Methods

### Human endometrial tissue samples

The study has been approved by Institutional Review Committee of Michigan State University, Greenville Health System and University of North Carolina, and written informed consent was obtained from all participants. All methods were performed in accordance with the relevant guidelines and regulations. The human endometrial samples were collected from Michigan State University’s Center for Women’s Health Research Female Reproductive Tract Biorepository (Grand Rapids, MI), the Greenville Hospital System (Greenville, SC), and the University of North Carolina (Chapel Hill, NC). Samples were collected as previously reported^22, 79, 80^. Briefly, we used eutopic endometrium derived from women with endometriosis and compared it to endometrium from women that did not have endometriosis. Subjects reported regular cycles and were between the ages of 18 and 45. We confirmed the presence of disease at the time of laparoscopy in the endometriosis group. Women who were laparoscopically negative for this disease were placed into the control group. For control eutopic endometrium, 21 samples were collected from the proliferative (n = 5) and secretory phase (n = 16) for Western blot analysis and 23 samples were collected from the proliferative (n = 6) and secretory (n = 17) phase for immunohistochemistry analysis. For endometriosis eutopic endometrium, 54 samples were collected from the proliferative (n = 16) and secretory (n = 38) phase for Western blot analysis and 57 samples were collected for immunohistochemistry analysis. Use of an intrauterine device (IUD) or hormonal therapies in the 3 months preceding surgery was exclusionary for this study. Histologic dating of endometrial samples was performed by a board certified pathologist (DPS).

### Animals and tissue collection

All the experimental mice were maintained in a designated animal care facility according to Michigan State University’s Institutional Guidelines for the care and use of laboratory animals. All animal procedures were approved by the Institutional Animal Care and Use Committee of Michigan State University. All animal experiments were performed in accordance with the relevant guidelines and regulations. *Kras* conditional activated mice were generated by crossing *Pgr* ^*cre*/+^ with LSL-*K-ras* ^*G12D*/+^ mice (*Pgr* ^*cre*/+^ LSL-*K-ras* ^*G12D*/+^)^35, 36^. For the study, female *control (*LSL-*K-ras* ^*G12D*/+^ and *Pgr* ^*cre*/+^) mice were used.

### Cytokine measurements

Plasma samples obtained from endometriosis patients and healthy controls were evaluated using a laser bead technology based commercial multiplex assay for the cytokine analysis by Eve Tech (Eve Technologies, Calgary, AB, Canada). Briefly, color-coded polystyrene beads were coupled with capture antibodies for each respective target cytokine. After washing twice with 100 µL of wash buffer, 50 µL of sample was added to each well. Following 1-hour incubation, wells were washed 3 times with 100 µL of wash buffer prior to adding 25 µL of detection antibody. 50 µL of streptavidin-PE was added to each well and was incubated for 10 minutes. Beads were re-suspended in 125 µL of assay buffer and the plate was read using Bio Plex 200 Suspension Array System. Fluorescent intensity signals in direct proportion to protein bound to specific analyte beads were analyzed. Observed concentration for each target analyte was calculated against standard curve regression.

### Baboon endometrium samples

The endometriosis baboon animal model is reviewed and approved by the Institutional Animal Care and Use Committees (IACUCs) of both the University of Illinois at Chicago and Michigan State University. Endometriosis is induced by intraperitoneal inoculation of menstrual endometrium on two consecutive menstrual cycles and harvested using laparotomy via endometriectomy from five female baboons as previously described^81^. Laparotomies were performed at 3, 9, and 15 months post-inoculation to harvest the eutopic endometrial tissues and these endometrial tissues were used for immunohistochemistry analysis.

### Western blot analysis

Western blot analyses were performed as described previously^82^. Briefly, eutopic endometrial tissues were lysed with lysis buffer (150 mM NaCl, 10 mM Tris-HCl (pH 7.4), 2.5 mM EDTA, 0.125% Nonidet P-40 (vol/vol), a protease inhibitor cocktail (Roche, Indianapolis, IN) and a phosphatase inhibitor cocktail (Sigma Aldrich, St. Louis, MO). Equal amounts of total protein (20 μg) were separated on SDS-polyacrylamide gel electrophoresis and transferred onto polyvinylidene difluoride membrane (Millipore Corp., Bedford, MA). Membrane was blocked with 0.5% Casein in phosphate buffered saline (PBS) and incubated with antibodies against SIRT1 (9475; Cell Signaling, Danvers, MA), BCL6 (561520; BD Pharmingen, San Jose, CA), and β-actin (sc1616; Santa Cruz Biotechnology, Santa Cruz, CA). Immunoreactivity was visualized by autoradiography and band intensity was determined by relative densitometry using ImageJ (National Institute of Health), and normalized against the bands obtained for β-actin.

### Immunohistochemistry and immunofluorescence analyses

Immunohistochemistry and immunofluorescence analysis were performed as previously described^22^. The paraffin-embedded endometrial tissues were blocked with 10% normal serum in PBS (pH 7.5) and then incubated with antibodies against SIRT1 (9475 for IHC and 8469 for IF, Cell Signaling), BCL6 (14895, Cell Signaling), KRAS (ab55391, Abcam) and GLI1 (sc20687; Santa Cruz Biotechnology). For immunohistochemistry, sections were incubated with secondary antibody conjugated to horseradish peroxidase (Vector Laboratories, Burlingame, CA). Immunoreactivity was detected using the Vectastain Elite DAB kit (DAB-Vector Laboratories, Burlingame, CA) and counterstained with hematoxylin. A semi-quantitative grading system (H-score) was used to compare the immunohistochemical staining intensities as previously described^83^. For immunofluorescence, the sections were exposed to primary antibodies overnight at 4 °C and secondary antibodies (Alexa Fluor 488-conjugated anti-rabbit IgG (Invitrogen, Grand Island, NY) and Alexa Fluor 594-conjugated anti-mouse IgG (Invitrogen) for 2 hour at room temperature. 4′,6‐diamidino‐2‐phenylindole (DAPI; Vector Laboratories) was used to enable nuclear visualization. The IgG antibody was intended for use as a negative control with SIRT1 and BCL6 proteins in the women endometrium (Supplementary Fig. S3).

### Immunoprecipitation analysis

Immunoprecipitation analysis were performed as previously described^80^. Protein lysates were immunoprecipitated with anti-SIRT1 (Cell Signaling) antibodies with protein A-agarose (Pierce Biotechnology, Rockford, IL) overnight at 4 °C. Immunocomplexes were subjected to Western blot analysis using anti-BCL6 (561520, BD Pharmingen) and anti-SIRT1 antibodies (Cell Signaling) antibodies.

### Cell culture and treatment

Ishikawa cells, epithelial cells of human endometrial adenocarcinoma, were maintained in Dulbecco’s Modified Eagle’s Medium with F12 (Gibco, Grand Island, NY) containing 10% fetal bovine serum (FBS; Gibco) and 1% penicillin streptomycin (P/S; Gibco) at 37 °C in 5% CO_2_. Ishikawa cells were pre-treated with 10 nM estradiol (E2, Sigma-Aldrich, St. Louis, MO) for 1 day and restored. After 2 days, these cells were treated with E2 + 1 μM medroxyprogesterone acetate (MPA; Sigma-Aldrich) and then incubated for the indicated time. All experiments were performed in triplicate.

### RNA isolation and quantitative real-time PCR

Total RNA was isolated from mouse uterine tissues or Ishikawa cell pellets using the RNeasy purification kit (Qiagen, Valencia, CA) according to the manufacturer’s instructions. Then, cDNA were synthesized using quantitative PCR random hexamers and MMLV Reverse Transcriptase (Invitrogen Crop., Carlsbad, CA). The expression levels of *GLI1* (TaqMan 00494654) were measured by quantitative real-time PCR using RT-PCR Universal Master Mix reagent (Applied Biosystems, Foster City, CA) according to the manufacturer’s instructions. mRNA quantities were normalized against the housekeeping gene, 18S RNA using ABI rRNA control reagents.

### Chromatinimmunoprecipitation (ChIP)

ChIP analysis was conducted by Active Motif (Carlsbad, CA) using Ishikawa cells treated with vehicle or E2 + MPA for 24 hours. ChIP assays were performed as previously described^80^. Briefly, 100 μg of chromatin from Ishkawa cells were immunoprecipitated by 4 μg of antibodies against BCL6 (BD Pharmingen). Eluted DNA was amplified with specific primers using SYBR Green Supermix (Bio-Rad Laboratories, Inc., Hercules, CA). Primers used in PCR were as follows: BCL6 A (forward: 5′-GTCCTGGGGGTGCAATAAG-3′; reverse: 5′-CCCCTCACCTCCCTTCTATT-3′), BCL6 B (forward: 5′-ACTGACCTTCCACACCCAAG-3′; reverse: 5′-GGAGGAAGCATGACAAGGAA-3′), and negative control (N.C.) (forward: 5′-CCTATCCCACCCCTTCACCA-3′; reverse: 5′-TAGCCTGCCCACCTCAGGAT-3′). The resulting signals were normalized to input activity.

### Statistical analysis

Statistical analyses were performed using the Student’s t-test for data with only two groups. For data containing more than two groups, we performed an analysis of variance (ANOVA) test and analyzed by Tukey or Bonferroni test for pairwise t-test. All data are presented as means ± SEM. p < 0.05 was considered statistically significant. All statistical analyses were performed using the Instat package from GraphPad (San Diego, CA).

## Electronic supplementary material

Supplemental Information
