# Supplementary material for: KRAS Activation and over-expression of SIRT1/BCL6 Contributes to the Pathogenesis of Endometriosis and Progesterone Resistance
Source: Sci Rep. 2017 Jul 28;7:6765. doi: 10.1038/s41598-017-04577-w (PMC5533722; doi:10.1038/s41598-017-04577-w)
Supplement: Supplementary file 1 — Supplemental Information [file 41598_2017_4577_MOESM1_ESM.pdf]

Supplementary Information

KRAS Activation and over-expression of SIRT1/BCL6 Contributes to the Pathogenesis of Endometriosis and Progesterone Resistance.

Jung-Yoon Yoo[1], Tae Hoon Kim[1], Asgerally T. Fazleabas[1, 2], Wilder A. Palomino[3], Soo Hyun Ahn[4], Chandrakant Tayade[4], David P. Schammel[5], Steven L. Young[6], Jae-Wook Jeong[1, 2, *], and Bruce A. Lessey[7, *]

[1] Obstetrics, Gynecology & Reproductive Biology, Michigan State University, Grand Rapids, MI 49503, USA

[2] Department of Women's Health, Spectrum Health System, Grand Rapids, MI 49341, USA

[3] Institute for Maternal and Child Research, Faculty of Medicine, University of Chile, Santiago, Chile

[4] Department of Biomedical and Molecular Sciences, Queens University, Kingston, ON K7L 3N6, Canada

[5] Pathology Associates, Greenville Hospital System, Greenville SC 29605, USA

[6] Obstetrics and Gynecology, University of North Carolina, Chapel Hill, NC 27514, USA

[7] Obstetrics and Gynecology, Greenville Health System, Greenville, SC 29605, USA

* Corresponding authors E-mail: blessey@ghs.org and jeongj@msu.edu

This PDF includes:

Supplementary Figure S1. Immunohistochemical analysis of KRAS and SIRT1 proteins in the endometrium during menstrual cycle in control women.

Supplementary Figure S2. Full-length Western blots of Fig. 2a.

Supplementary Figure S3. Immunofluorescence analysis of IgG antibody intended for use as a negative control with SIRT1 and BCL6 proteins in the women endometrium.

Supplementary Figure S4. Full-length Western blots of Fig. 3c.

Supplementary Figure S5. Full-length Western blots of Fig. 6a.

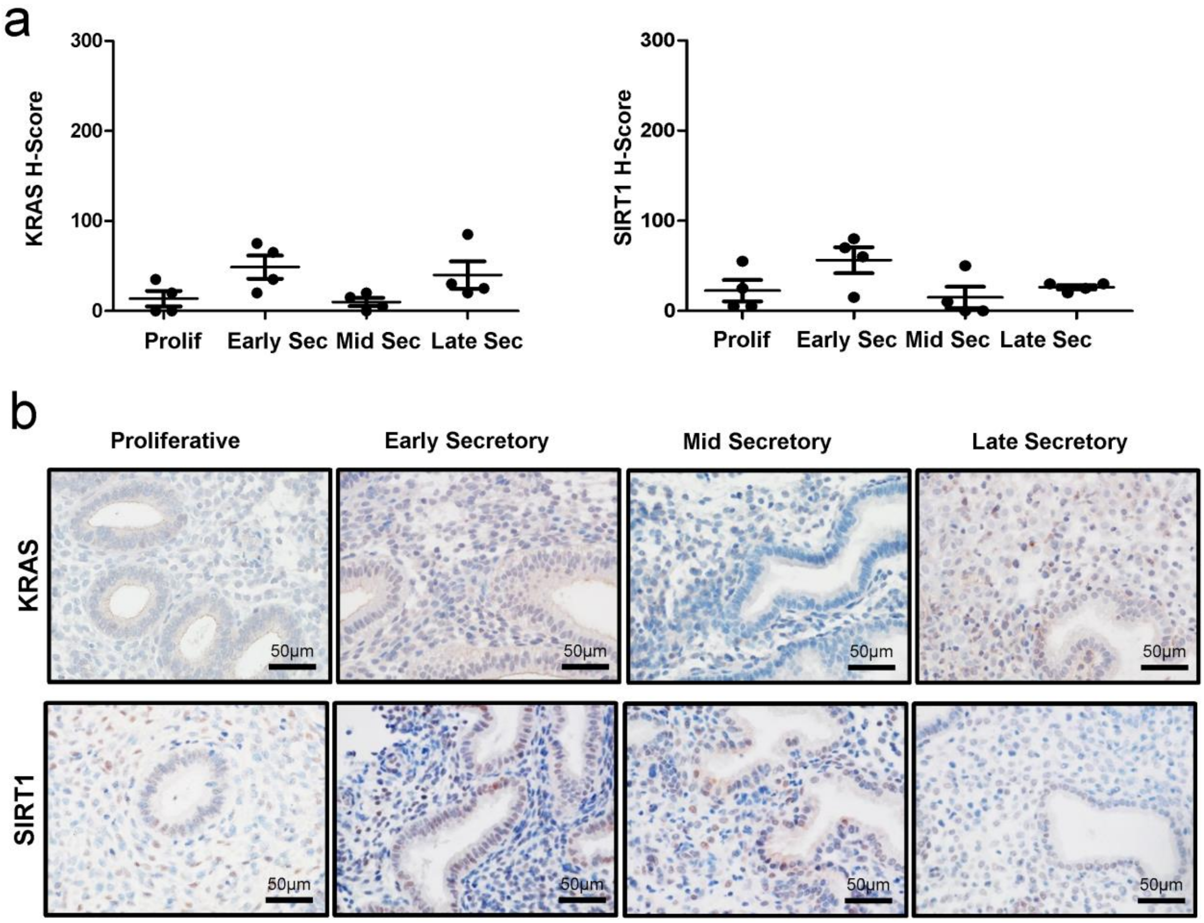

Supplementary Figure S1. Immunohistochemical analysis of KRAS and SIRT1 proteins in the endometrium during menstrual cycle in control women. (a and b) H-score (a) and representative photomicrograph of immunohistochemical staining (b) of KRAS and SIRT1 expression in proliferative and early, mid, and late secretory phase of the menstrual cycle.

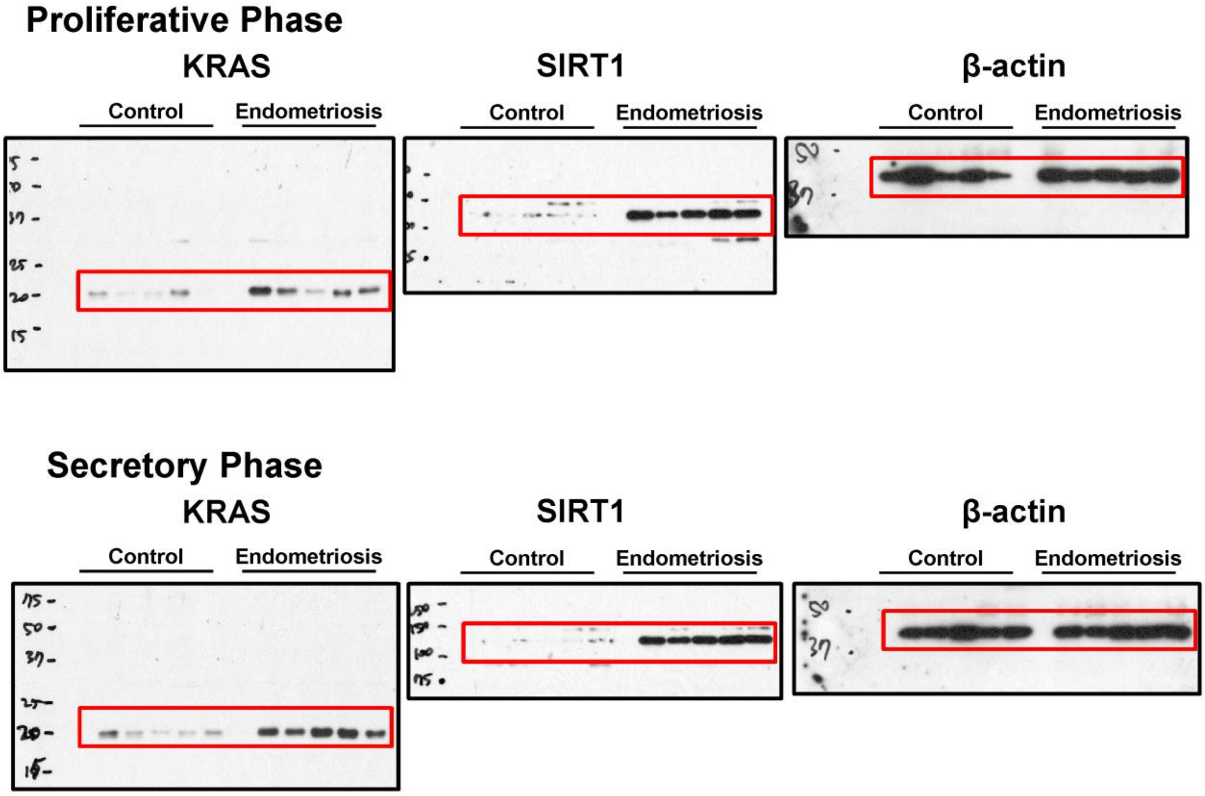

Supplementary Figure S2. Full-length Western blots of Fig. 2a.

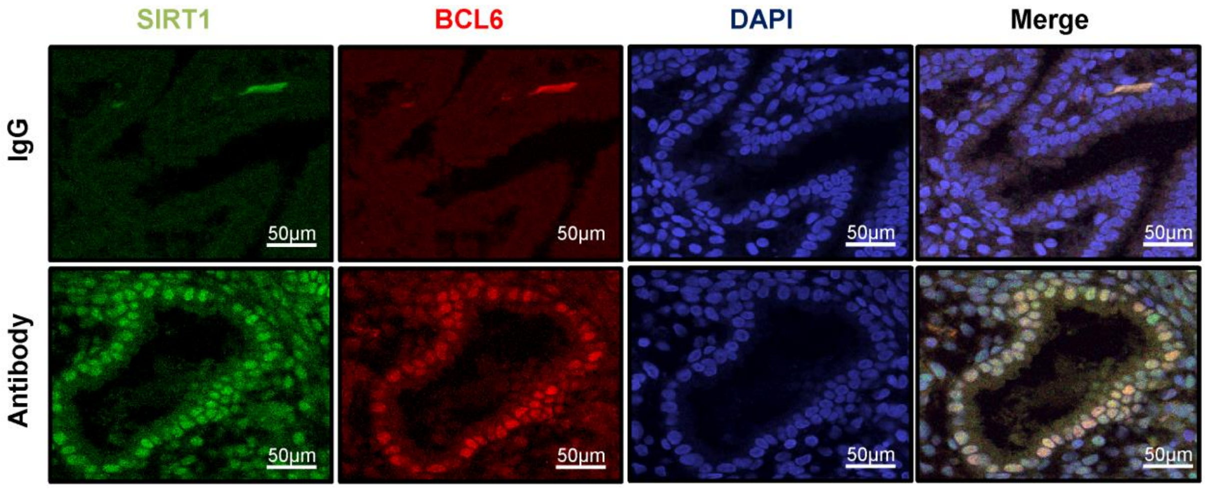

Supplementary Figure S3. Immunofluorescence analysis of IgG antibody intended for use as a negative control with SIRT1 and BCL6 proteins in the women endometrium.

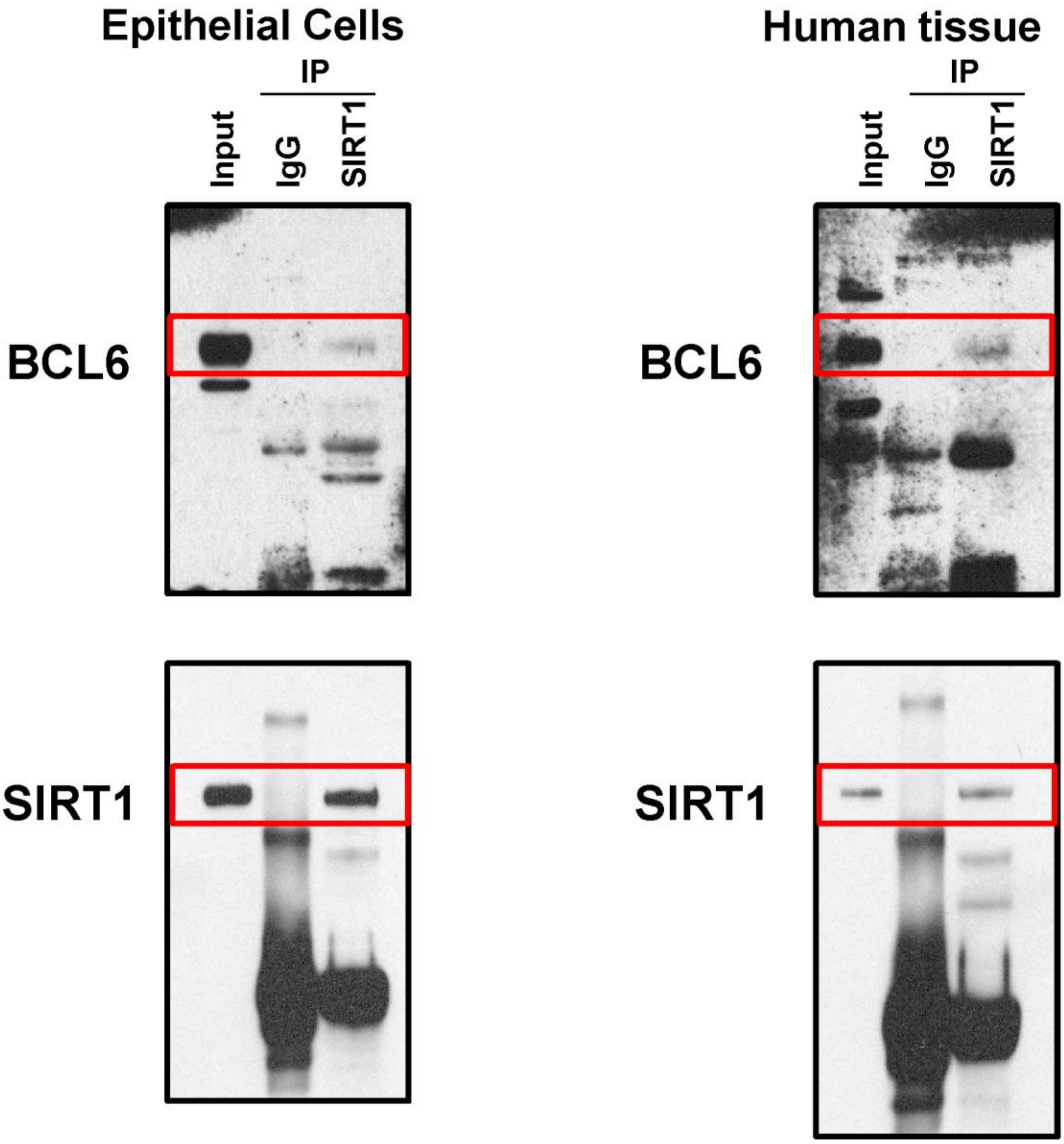

Supplementary Figure S4. Full-length Western blots of Fig. 3c.

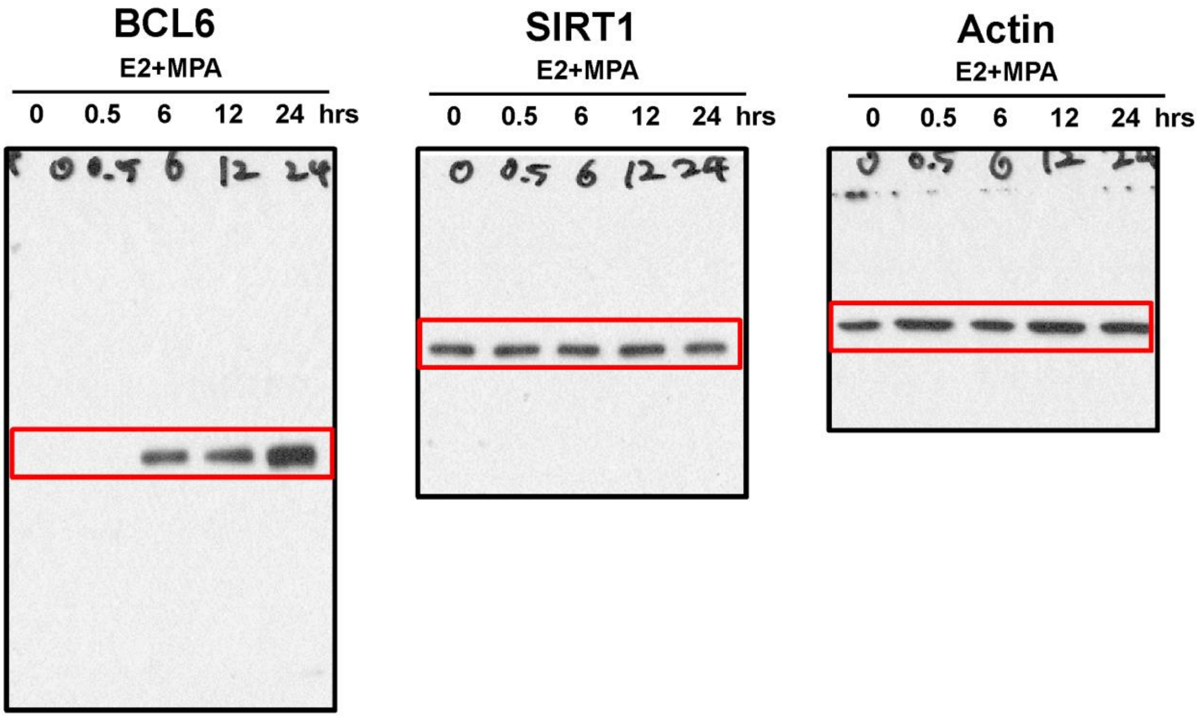

Supplementary Figure S5. Full-length Western blots of Fig. 6a.
